# Disseminated *Mycobacterium simiae* infection in a patient with adult-onset immunodeficiency due to anti-interferon-gamma antibodies – a case report

**DOI:** 10.1186/s12879-020-04984-x

**Published:** 2020-04-01

**Authors:** B. S. D. P. Keragala, C. N. Gunasekera, P. D. Yesudian, Chandima Guruge, B. S. Dissanayaka, D. P. Liyanagama, G. I. M. Jinadasa, S. R. Constantine, H. M. M. T. B. Herath

**Affiliations:** 1 National Hospital of Sri Lanka, Colombo, Sri Lanka; 2 Wrexham Maelor Hospital, Wrexham, UK

**Keywords:** Disseminated *Mycobacterium simiae* infection, Adult-onset immunodeficiency due to anti-interferon-gamma antibodies, Sri lanka, Case report

## Abstract

**Background:**

Mycobacterial species other than *Mycobacterium tuberculosis* and *Mycobacterium leprae* are generally free-living organisms and *Mycobacterium simiae* is one of the slowest growing Non-tuberculous mycobacteria. This is the first case report of *Mycobacterium simiae* infection in Sri Lanka and only very few cases with extrapulmonary manifestation reported in the literature.

**Case presentation:**

A 24-year-old, previously healthy Sri Lankan male presented with generalized lymphadenopathy with discharging sinuses, evening pyrexia, weight loss, poor appetite and splenomegaly. Lymph node biopsies showed sheets of macrophages packed with organisms in the absence of granulomata. Ziehl Neelsen, Wade Fite and Giemsa stains revealed numerous red coloured acid-fast bacilli within foamy histiocytes. Slit skin smear for leprosy was negative and tuberculosis, fungal and bacterial cultures of the lymph node and bone marrow did not reveal any growth. Later he developed watery diarrhea and colonoscopy revealed multiple small polyps and ulcers throughout the colon extending up to the ileum, Which was confirmed to be due to cytomegalovirus confirmed by PCR and successfully treated with ganciclovir. Positron emission tomography scan guided biopsies of the gut and lymph nodes confirmed presence of mycobacterial spindle cell pseudo-tumours and PCR assays revealed positive HSP65. The culture grew *Mycobacterium Simiae*. Flow cytometry analysis on patient’s blood showed extremely low T and B cell counts and immunofixation revealed low immunoglobulin levels. His condition was later diagnosed as adult onset immunodeficiency due to anti- interferon – gamma autoantibodies. He was initially commenced on empirical anti-TB treatment with atypical mycobacterial coverage. He is currently on a combination of daily clarithromycin, ciprofloxacin, linezolid with monthly 2 g/kg/intravenous immunoglobulin to which, he had a remarkable clinical response with complete resolution of lymphadenopathy and healing of sinuses.

**Conclusions:**

This infection is considered to be restricted to certain geographic areas such as mainly Iran, Cuba, Israel and Arizona and this is the first case report from Sri lanka. Even though the infection is mostly seen in the elderly patients, our patient was only 24 years old. In the literature pulmonary involvement was common presentation, but in this case the patient had generalized lymphadenopathy and colonic involvement without pulmonary involvement.

## Background

Mycobacterial species other than *Mycobacterium tuberculosis* and *Mycobacterium leprae* are generally free-living organisms that are found in water, soil, domestic and wild animals, milk, and food and has been noted in environment as viable by Reverse transcription polymerase chain reaction [1, 2].

*Mycobacterium simiae* is one of the slowest growing Non-tuberculous mycobacteria (NTM) which was initially identified from rhesus monkeys in 1965 [3] and was reported in the Southern United States, Cuba, Palestine, Iran, Israel, Turkey, and Japan [4]. Here we report a case of a young man who initially presented with constitutional symptoms like lymphadenopathy, splenomegaly and later intestinal lesions and was diagnosed to be infected with *Mycobacterium simiae*. He was also diagnosed with adult onset immunodeficiency with anti- interferon – gamma autoantibodies. Adult onset immunodeficiency with anti-interferon-gamma autoantibodies is a rare acquired condition where high titres of neutralizing anti-Interferon gamma antibodies affecting Interferon gamma-Interleukin 12 pathways are detected. Typical cases are young, previously healthy individuals of Asian origin presenting with lymphadenopathy (cervical or generalized), constitutional symptoms and reactive skin changes due to disseminated non-tuberculous mycobacterial infections. This is the first case report of *Mycobacterium simiae* infection in Sri Lanka and only very few cases of extrapulmonary cases reported in the literature.

## Case presentation

**Fig. 1:**
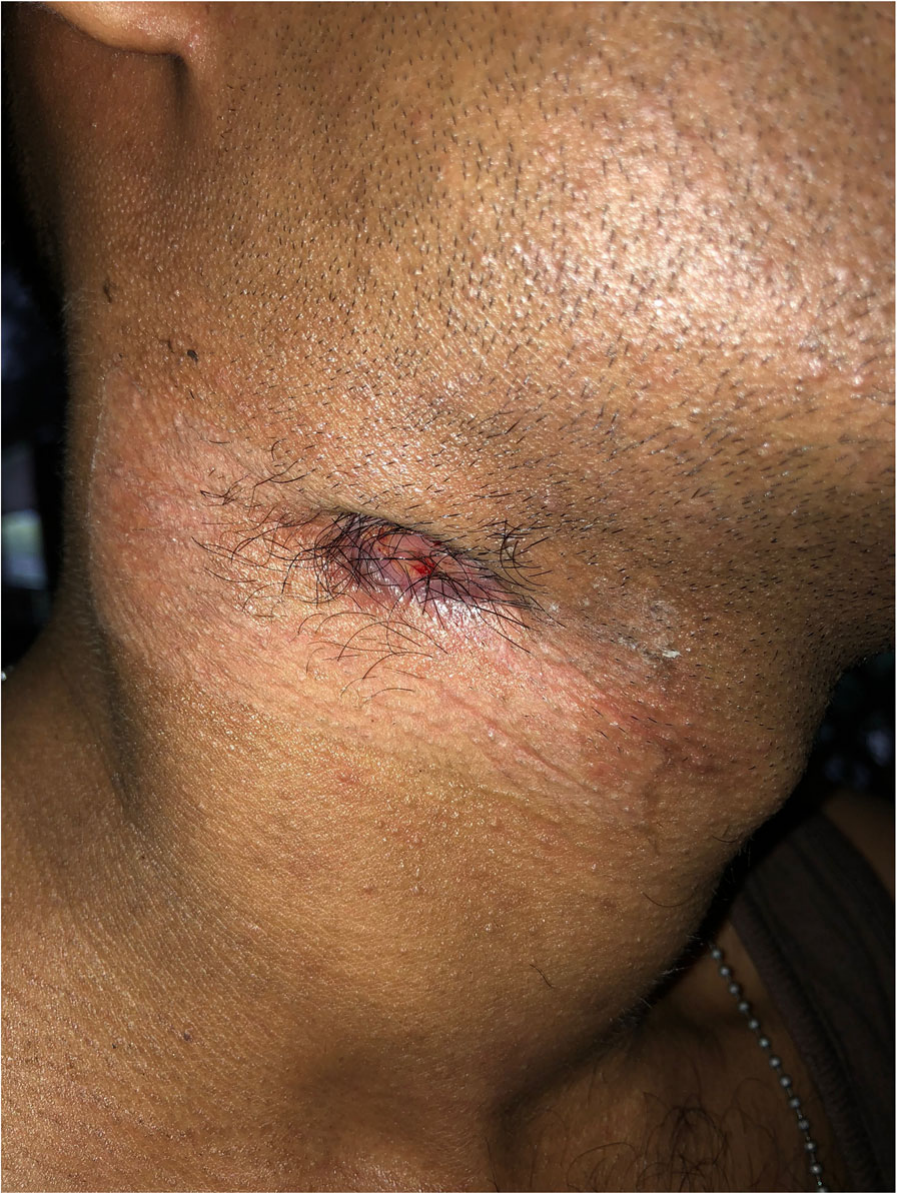
Cervical and axillary lymph nodes later developed in to discharging sinuses

**Fig. 2:**
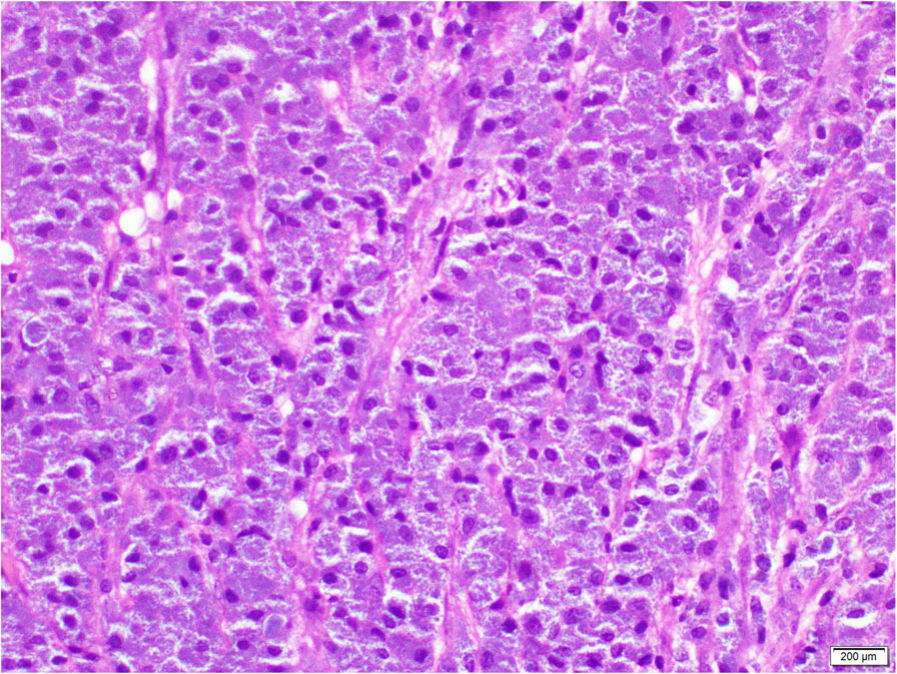
Repeated lymph node biopsies showing sheets of macrophages packed with organisms in the absence of granulomata

**Fig. 3:**
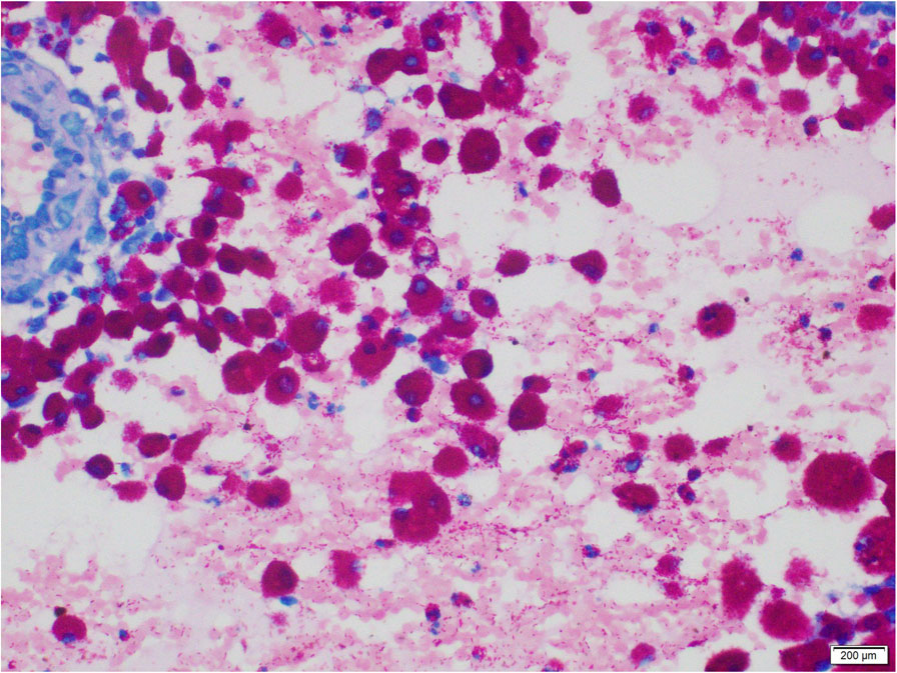
Ziehl Neelsen revealing numerous red coloured acid-fast bacilli within foamy histiocytes

**Fig. 4:**
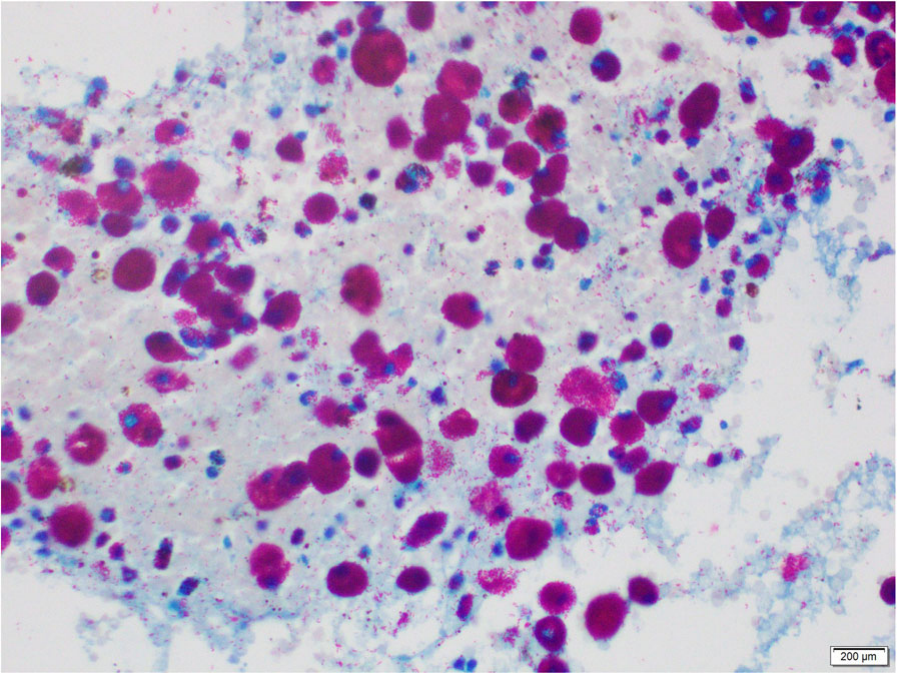
Wade Fite revealed numerous red coloured acid-fast bacilli within foamy histiocytes

**Fig. 5:**
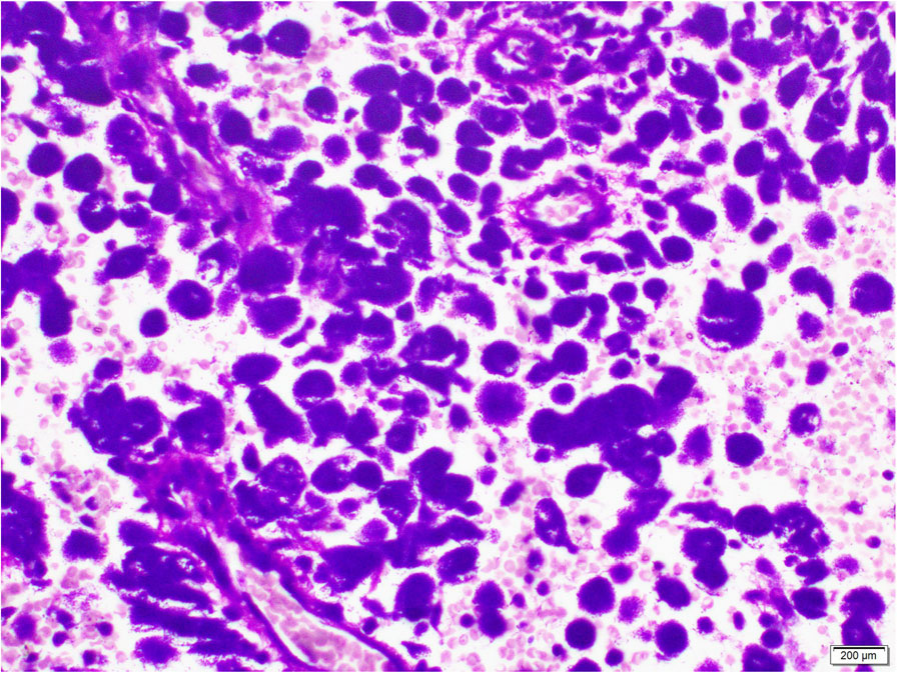
Giemsa stains revealed numerous red coloured acid-fast bacilli within foamy histiocytes

**Fig. 6:**
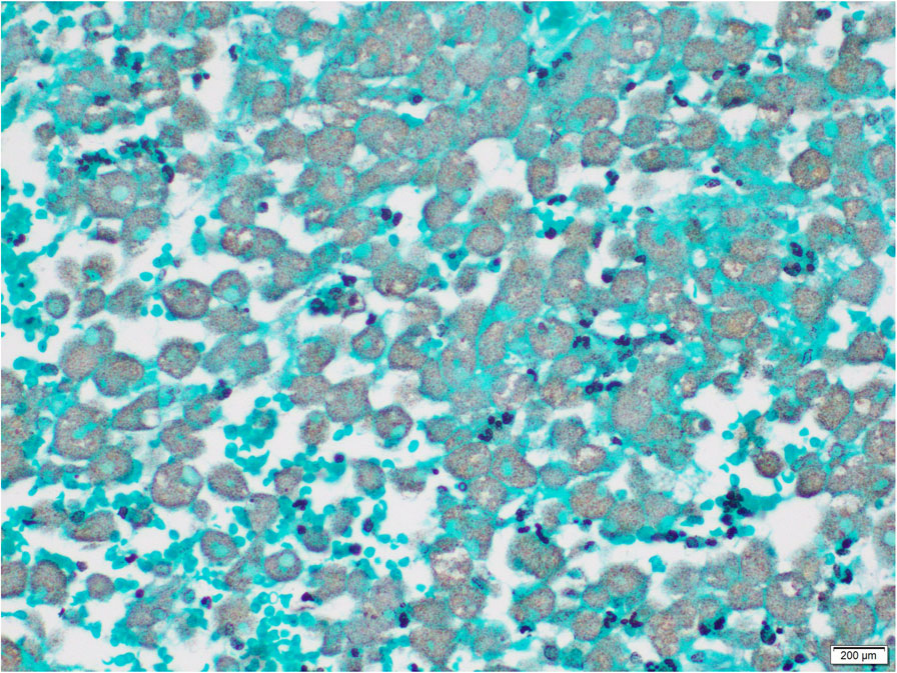
Grocott stain showing weak positivity

A 24-year-old, previously healthy Sri Lankan male was referred for evaluation of generalized lymphadenopathy. He also had evening pyrexia, weight loss, poor appetite and splenomegaly. Cervical and axillary lymph nodes later developed in to discharging sinuses (Fig. 1). Repeated lymph node biopsies showed sheets of macrophages packed with organisms in the absence of granulomata (Fig. 2). Ziehl Neelsen (Fig. 3), Wade Fite (Fig. 4) and Giemsa stains (Fig. 5) revealed numerous red coloured acid-fast bacilli within foamy histiocytes. Grocott stain (Fig. 6) was weakly positive. However Slit skin smear for leprosy was negative and tuberculosis (TB), fungal and bacterial cultures of the lymph node and bone marrow did not reveal any growth. Tuberculosis interferon gamma release assays and TB Polymerase chain reaction (PCR) were also negative. Computerized tomography (CT) scan showed splenomegaly and multiple paraaortic and inguinal lymphadenopathy. Antinuclear antibodies were negative and HIV (human immunodeficiency virus) 1 and 2 antibodies and P24 antigen were 2 times negative in 3 months apart. Due to the unusual presentation, the patient was investigated for possible immunodeficiency with flowcytometry analysis, which showed extremely low T and B cell counts and immunofixation revealed low immunoglobulin levels (Table 1).

**Table 1:** Low T and B cell counts and low immunoglobulin levels

Investigation	Result	Reference value
CD4 lymphocyte absolute counts	45 cells/mm3	300–1400
CD8 lymphocyte absolute counts	21 cells/mm3	200–900
CD4 / CD8 ratio	2.95	0.7–2.5
CD3 lymphocyte absolute counts	72 cells/mm3	700–2100
CD19 lymphocyte absolute counts	43 cells/mm3	100–500
Ig E level	13.9 IU/mL	20.4 (95% ile = 87)
IgA level	56.0 mg/dL	70–400
IgG level	670 mg/dL	700–1600
IgM level	70.5 mg/dL	40–230

**Fig. 7:**
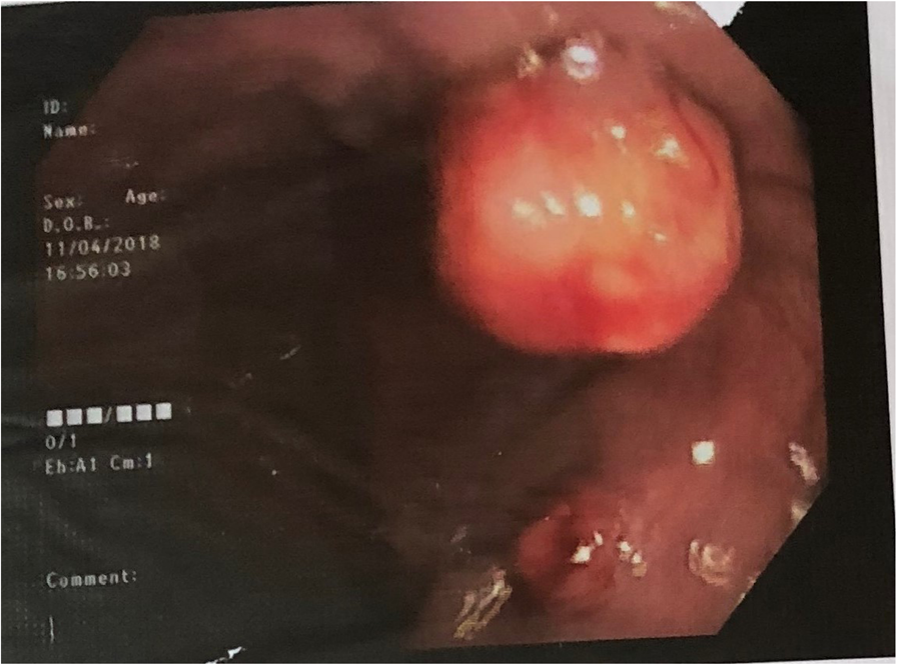
Colonoscopy revealed multiple small polyps and ulcers throughout the colon extending up to the ileum

**Fig. 8:**
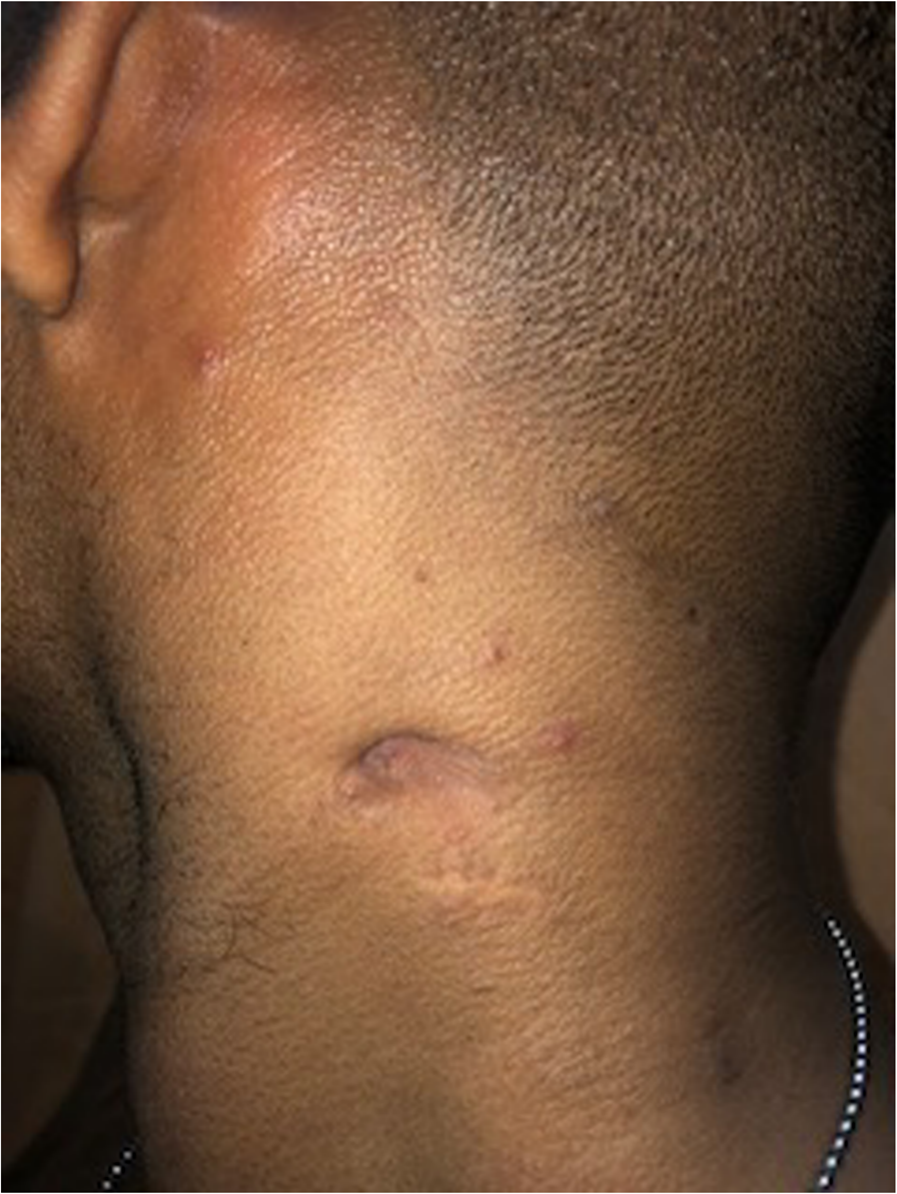
Complete resolution of lymphadenopathy and healing of sinuses

**Fig. 9:**
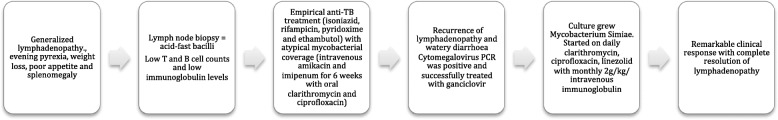
Time line of the case report

As the condition deteriorated, he was commenced on empirical anti-TB treatment (isoniazid, rifampicin, pyridoxime and ethambutol) with atypical mycobacterial coverage (intravenous amikacin and imipenum for 6 weeks with oral clarithromycin and ciprofloxacin). Although he responded initially, 8 months into treatment he relapsed with recurrence of lymphadenopathy and watery diarrhoea. Colonoscopy revealed multiple small polyps and ulcers throughout the colon extending up to the ileum (Fig. 7), which was confirmed to be *cytomegalovirus* by PCR and successfully treated with ganciclovir. Positron emission tomography scan guided biopsies of the gut and lymph nodes confirmed mycobacterial spindle cell pseudo-tumours. Then we sequenced PCR products from the lymph node biopsies and IS6110 PCR assay was negative and and HSP65 PCR assay was positive. IS*6110* is uniquely found in *Mycobacterium tuberculosis* complex [5] and hsp65 is positive in *Mycobacterium tuberculosis* complex and nontuberculosis mycobacteria [6]. Later the culture grew *Mycobacterium Simiae*. After seeking advice from an expert centre, his condition was later diagnosed as adult onset immunodeficiency due to anti- interferon – gamma autoantibodies. Anti- interferon – gamma autoantibodies were measured by Enzyme-Linked Immunosorbent Assay (ELISA) method after serially diluting samples from the patient and control and was positive was positive at 1:30000. He is currently on a combination of daily clarithromycin, ciprofloxacin, linezolid with monthly 2 g/kg/intravenous immunoglobulin (IVIG) treatment, to which, he had a remarkable clinical response with complete resolution of lymphadenopathy and healing of sinuses (Fig. 8). The immunoglobulin levels returned to normal levels, except Ig A fraction and CD4 counts improved (Table 2). Immuno suppressants or biologics (Rituximab) were not considered due to the fear of dissemination of infection. Time line is given in the Fig. 9.

**Table 2:** T and B cell counts and low immunoglobulin levels after treatment

Investigation	Result	Reference range
CD4 lymphocyte absolute counts	160 cells/mm3	500–1500
CD8 lymphocyte absolute counts	54 cells/mm3	200–900
Ig E level	13.9 IU/mL	20.4
IgA level	57.0 mg/dL	70–400
IgG level	1079 mg/dL	700–1600
IgM level	84 mg/dL	40–230

## Discussion and conclusions

*M. simiae* is a frequent colonizer of the lung and is not usually pathogenic in immunocompetent people. This infection seems to be restricted to certain geographic areas such as mainly Iran, Cuba, Israel and Arizona most probably due to common environmental factors including temperature and humidity [7, 8] However *M. simiae* has also been isolated in in many distant countries as well [3]. In a recent study from India, *M. simiae* bacilli were closely associated with *M.leprae* and other pathogenic non-tuberculous mycobacteria in leprosy endemic area due to water and soil sources [9]. The infection is mostly seen in the elderly patients [3] and in patients with diabetes mellitus, acquired immunodeficiency syndrome (AIDS) [10], cardiovascular disease, chronic lung diseases and malignancies [11]. The average age was 61 years in one case series. However several case reports describe lymphadenopathy in children caused by *M. simiae* as well [12]. Most common symptoms reported in these patients constitutional symptoms with sweating, weight loss, low-grade fever and pulmonary symptoms such as productive cough and hemoptysis [10, 11] Only very few cases of extrapulmonary *M. simiae* infections are found the literature including vertebral osteomyelitis [13], localized lymphadenitis [14], genitourinary tract infection [15, 16] skin lesions [14], parotid gland infection [13] and nervous system infection [17]. In our patient the main presenting complains were fever, constitutionals symptoms with generalized lymphadenopathy and splenomegaly. Later he developed blood and mucous diarrhea due to colonic involvement. Interestingly our patient did not have respiratory system involvement and high resolution CT of the chest and bronchoalveloar larvage were normal. The histopathological findings in *M. simiae* infection can be classical tuberculous-like granulomas with varying degrees of necrosis or non-necrotic granulomas [18]. In our patient biopsy of the lymph node revealed large nodular collection of epitheliod histiocytes and scatted small collections of foamy histiocytes. No granulomas or caseous necrosis was seen and both Wade- Fite stain and Ziehl – Neelson stain revealed numorous red coloured acid-fast bacilli filling the cytoplasm of foamy histiocytes. Even though interferon-gamma release assays was negative in our patient in many patients with disseminated non-tuberculous mycobacteria infection and neutralizing anti- interferon-gamma autoantibodies, interferon-gamma release assays were indeterminate because of extremely low or undetectable interferon-gamma levels in the mitogen tubes [19].

No firm conclusions regarding the best regimen and duration of treatment is available due to limited case reports and series. In our patient we initially started anti tuberculosis treatment (Isoniacid, ethambutol, pyrazinamide and rifampicin). Later he was also given intravenous amikacin and imipenum for 6 weeks with oral clarithromycin and ciprofloxacin. A resent retrospective study suggested clarithromycin in different combinations with cotrimoxazole, moxifloxacin, and amikacin for a duration of 6 to 24 months [11]. Van Ingen J et al., suggested clarithromycin combined with moxifloxacin and another susceptible drug (e.g. clofazimine, Trimethoprim (TMP) / Sulfamethoxazole (SMX), amikacin, streptomycin) [20]. Adding clofazimine to amikacin can have synergistic activity against *M. simiae* [21].Another study suggested a combination of four-drug regimen containing clarithromycin, ethambutol, rifabutin and streptomycin [22]. However because of variable susceptibly profile of this pathogen in different geographic locations susceptibility testing is important before initiating therapy [11]. Unfortunately drug susceptibility testing was not available in our setup. We planned out our treatment and follow up depending on the available literature in treating this infection. Our patient initially responded to anti-TB treatment with amikacin, clarithromycin and ciprofloxacin but later relapsed may be due to development of resistance. However adding linezolid and immunoglobulins led to a remarkable clinical improvement. Linezolid was active against 100% of isolates of *M.simiae* in one study [23].

Adult-onset immunodeficiency with anti-interferon-gamma autoantibodies is an immunodeficiency disorder, which is associated with susceptibility to disseminated infections such as non-tuberculous mycobacteria, non-typhoidal salmonella, *cytomegalovirus, Penicillium marneffei*, and *varicella zoster* virus [24, 25] Our patient initially got a mycobacterial infection and later developed *cytomegaloviral* infection. This needs to be considered in HIV negative adult patients with unknown immunodeficiency and severe, persistent or recurrent infections especially of Asian origin and having reactive skin lesions and autoimmune endocrinopathy. Unusual presentations and atypical staining patterns warrant investigation for immunodeficiency status and associated infections with atypical organisms. Though the CD4 count was extremely low in our patient, which was supportive of idiopathic CD4 lymphocytopenia, it is uncommon to have a good CD4 to CD8 ratio > 0.8. Hence, adult onset immunodeficiency with anti- interferon – gamma autoantibodies was suspected. However in most patients with adult-onset immunodeficiency due to anti-inferferon-gamma antibodies have normal CD 4 counts [26]. The initial low counts would have been due on going sepsis and after the infection is controlled the counts improved. Currently there is no standard treatment for adult onset immunodeficiency with anti-interferon-gamma autoantibodies but long-term antimicrobial therapy and rituximab have been used with variable success [19]. Immunosuppressants were not considered in our patient due to the fear of dissemination of infection. He remains in remission with the use of monthly 2 g/kg IVIG, clarithromycin, ciprofloxacin and linezolid. Even though IVIG was originally used as a replacement therapy for primary and secondary immunodeficiency diseases characterized by the absence or deficiency of antibody production there are other clinical benefits of IVIG treatment. Many of these other uses because of the anti-inflammatory and immunomodulatory effects of IVIG [27]. IVIG was also used in a patient with anti- interferon – gamma autoantibodies to suppress the production of autoantibodies but had had little efficacy in suppressing the production of autoantibodies in vitro testing [28]. It has been suggested that IVIG may be an alternative option in these patients due to its immunomodulatory effect [29]. In our patient, the affectivity of IVIG was subsequently was evidenced objectively by the improvement in CD4 cell count and immunoglobulin levels and subjectively by the clinical improvement.

This case in interesting because this is the first case report of *M.simiae* from Sri lanka and even though the infection is mostly seen in the elderly patients, our patient was only 24 years old. In the literature pulmonary involvement was the comment presentation, but in this case the patient had generalized lymphadenopathy and colonic involvement without pulmonary involvement. This case also highlights the importance of looking for an underlying immunodeficiency syndrome in patients with atypical infections. Drug susceptibility testing for atypical infections will be useful because of development of resistance.

## Data Availability

The datasets supporting the conclusions of this article are included within the article.

## Abbreviations

NTM: Non-tuberculous mycobacteria
TB: Tuberculosis
IVIG: Intravenous immunoglobulin
AIDS: Acquired immunodeficiency syndrome
